# Thermal Strain and Microstrain in a Polymorphic Schiff Base: Routes to Thermosalience

**DOI:** 10.3390/molecules30122567

**Published:** 2025-06-12

**Authors:** Teodoro Klaser, Marko Jaklin, Jasminka Popović, Ivan Grgičević, Željko Skoko

**Affiliations:** 1 Physics Department, Faculty of Science, University of Zagreb, Bijenička Cesta 32, 10000 Zagreb, Croatiamarko.jaklin@usc.es (M.J.); 2 Department of Physics, University of Trento, Via Sommarive 14, 38123 Trento, Italy; 3 Centro Singular de Investigación en Tecnoloxías Intelixentes, Rúa de Jenaro de la Fuente Domínguez, 15782 Santiago de Compostela, Spain; 4 Ruđer Bošković Institute, Bijenička Cesta 54, 10000 Zagreb, Croatia; jpopovic@irb.hr; 5 Labtim Adria d.o.o., Jarušisca 7A, 10200 Zagreb, Croatia; ivan.grgicevic@labtim.hr

**Keywords:** thermomechanical actuation, negative thermal expansion (NTE), variable-temperature X-ray powder diffraction (VT-XRPD), principal axis strain analysis, polymorphic transition dynamics, crystallographic stress anisotropy

## Abstract

We present a comprehensive structural and thermomechanical investigation of *N*-salicylideneaniline, a Schiff base derivative that exhibits remarkable thermosalient phase transition behavior. By combining variable-temperature X-ray powder diffraction (VT-XRPD), differential scanning calorimetry (DSC), hot-stage microscopy, and Hirshfeld surface analysis, we reveal two distinct thermosalient mechanisms operating in different polymorphic forms. Form I displays pronounced anisotropic thermal expansion with negative strain along a principal axis, culminating in a sudden and explosive phase transition into Form IV. In contrast, Form III transforms more gradually through a microstrain accumulation mechanism. Fingerprint plots and contact evolution from Hirshfeld surface analysis further support this dual-mechanism model. These insights highlight the importance of integrating macro- and microscale structural descriptors to fully capture the mechanical behavior of responsive molecular solids. The findings not only enhance the fundamental understanding of thermosalience but also inform the rational design of functional materials for actuating and sensing applications.

## 1. Introduction

Thermosalient materials, commonly known as “jumping crystals,” represent a fascinating class of crystalline solids characterized by their unique ability to exhibit pronounced mechanical responses when subjected to temperature changes. Initially reported by Etter and Siedle in 1983 through their observations on (Phenylazaphenyl)-Pd-(hexafluoroacetonate) complexes, the thermosalient effect has since evolved into a highly intriguing phenomenon attracting significant scientific interest [1]. These materials convert thermal energy efficiently into mechanical work, leading to rapid and often spectacular physical actions such as jumping, twisting, or exploding [1,2,3,4,5,6,7,8,9,10,11,12,13,14,15,16,17,18,19,20,21,22,23]. Over the decades, considerable progress has been made in understanding the underlying mechanisms of this intriguing phenomenon. Current knowledge suggests that thermosalient behavior is often associated with sharp, rapid phase transitions accompanied by substantial anisotropic structural changes at the molecular level [13,14,24]. It has been established that many thermosalient crystals exhibit negative thermal expansion (NTE), an unusual behavior where at least one crystallographic direction contracts upon heating, accompanied by substantial positive expansion along perpendicular axes [4,14,25]. Furthermore, such phase transitions are typically isosymmetric and topotactic, involving slight molecular rearrangements that lead to significant changes in cell parameters, often exceeding 10% [14].

The classification proposed by Naumov’s group divides thermosalient materials into three classes based on hydrogen-bonding characteristics: Class I, molecules lacking strong hydrogen-bond donor and acceptor groups; Class II, containing hydrogen-bonding groups in sterically crowded environments; and Class III, featuring robust hydrogen-bonding functional groups [2,12,14,17]. This classification has provided valuable insights, driving the exploration of various molecular frameworks capable of demonstrating the thermosalient effect.

The potential applications of thermosalient materials span diverse technological and biomedical fields, reflecting their ability to convert energy into mechanical work efficiently. Proposed and demonstrated applications include components for smart medical devices, actuators, artificial muscles, biomimetic devices, dynamic elements in electronics, heat-sensitive sensors, and even thermally activated switches or fuses [8,10,11,14,18,21,26]. The versatility and adaptability of these materials continue to inspire research aimed at refining their functional performance and exploring new avenues for application.

Reversible polymorphic phase transitions have been widely studied in molecular and functional materials for their implications on tunable properties and stability. For instance, Ba_3_Mg_3_(BO_3_)_3_F_3_ exhibits a thermally driven α–β phase transformation that modulates its nonlinear optical response, making it attractive for UV–optical applications despite the absence of mechanical actuation [27]. Similarly, AZD5462, a pharmaceutical compound, undergoes a reversible single-crystal-to-single-crystal transition between Forms A and G, associated with conformational changes and enantiotropic relationships, as revealed by in situ thermal and diffraction techniques [28]. Although these systems do not exhibit thermosalient effects, they illustrate the significance of understanding the structural evolution preceding polymorphic transformations—an approach we adopt here to uncover the mechanistic origins of mechanical responsiveness in our system.

*N*-salicylideneaniline and its derivatives have attracted notable attention due to their pronounced polymorphism and intriguing mechanical responses. Mittapalli et al. (2017) reported the mechanochemical synthesis of dichloro derivatives of *N*-salicylideneaniline, highlighting their thermosalient behavior across multiple polymorphs [29]. Specifically, they described three polymorphic forms exhibiting diverse mechanical behaviors—jumping for Forms I and III, and explosive responses for Form II upon thermal stimulation. These phenomena were attributed primarily to differences in crystal packing and weak intermolecular interactions, notably involving halogen bonding (C—Cl···O) and amide hydrogen bonding [29]. The research emphasized the crucial role played by crystal morphology and anisotropic stress distribution in mediating the mechanical responses of salicidene derivatives. The detailed crystallographic study established that variations in halogen substituents significantly influenced the strength and nature of halogen bonds, thereby modulating thermal responsiveness. Mittapalli’s work laid essential groundwork for further exploration of the relationship between molecular interactions, crystal structure, and mechanical responses in this class of Schiff base derivatives.

In the present study, we advance the understanding of thermosalient behavior in *N*-salicylideneaniline through a comprehensive suite of experimental techniques, including variable-temperature X-ray powder diffraction (VTXRPD), differential scanning calorimetry (DSC), and hot-stage microscopy, supported by anisotropic thermal expansion analysis using PASCal (v2.2.0), microstrain evaluation from XRD line broadening, and detailed Hirshfeld surface and fingerprint plot analysis. These methods reveal multiple phase transitions between polymorphs, each accompanied by distinct mechanical responses. Crucially, our results demonstrate that the thermosalient effect in this system arises from two fundamentally different mechanisms: an abrupt, anisotropic strain-driven transition in Form I, and a more subtle, microstrain accumulation mechanism in Form III. This dual behavior challenges the traditional view that thermosalient transitions are primarily governed by large-scale lattice parameter discontinuities and instead emphasizes the importance of local interaction dynamics and contact flexibility. Detailed crystallographic analysis reveals that the transitions are strongly correlated with extraordinary anisotropic thermal expansion, particularly in Form I, which exhibits sharp changes in lattice dimensions along specific crystallographic directions. In contrast, Form III maintains continuous lattice evolution while exhibiting increasing microstrain and contact compression—factors that ultimately culminate in mechanical motion. The structural relaxation observed in the high-temperature Form IV further supports this model, reflecting a thermodynamically stable, strain-free endpoint. By elucidating how both macroscopic anisotropic strain and distributed microstrain can independently lead to thermosalient actuation, our findings offer a new framework for interpreting mechanical responsiveness in molecular crystals. To our knowledge, this is the first example of a polymorphic system in which two essentially different mechanisms independently give rise to the thermosalient effect: one form actuates through quasi-martensitic lattice strain release, while another exhibits actuation via microstrain accumulation. This work underscores the critical role of thermal expansion behavior in determining structural reactivity and provides practical insight into the design of responsive crystalline materials with tunable actuation profiles.

## 2. Results and Discussion

**Figure 1:**
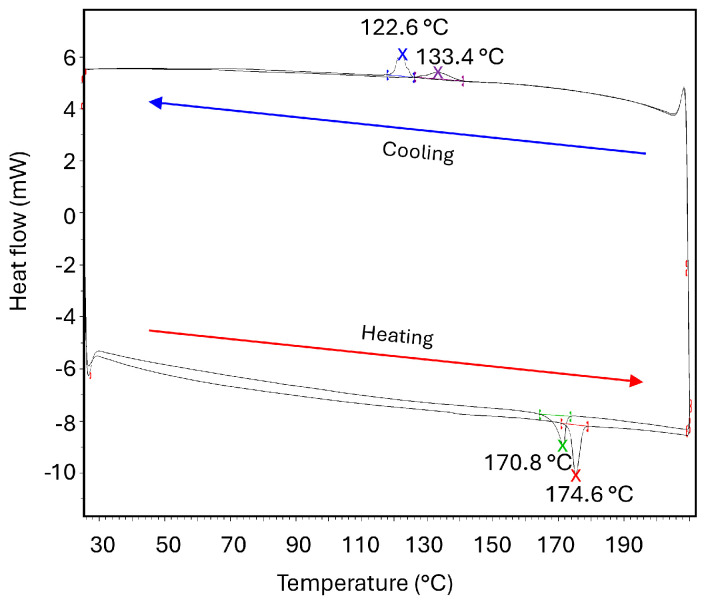
DSC thermogram of *N*-salicylideneaniline aniline over two heating and cooling cycles. A sharp endothermic peak near 453 K (~180 °C) corresponds to a thermosalient phase transition. Cooling peaks at 396 K (~123 °C) and 406 K (~133 °C) (first and second cycles, respectively) indicate recrystallization with thermal hysteresis and altered nucleation behavior after thermal cycling.

The thermal behavior of *N*-salicylideneaniline was systematically investigated by differential scanning calorimetry (DSC) using two consecutive heating and cooling cycles in the temperature range of 300–483 K (~27–210 °C). The resulting DSC curves are shown in Figure 1. Two cycles were performed to establish the reproducibility of the results. The obtained DSC thermogram clearly reveals distinct thermal events, which correspond to polymorphic transitions accompanied by noticeable heat exchanges.

**Figure 2:**
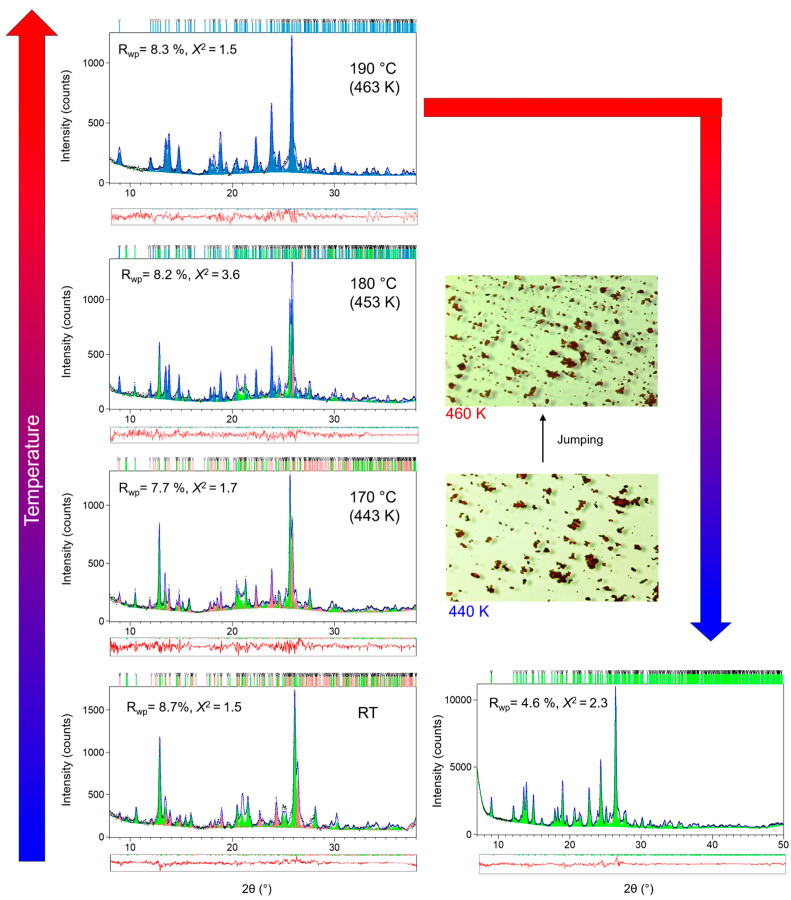
Le Bail fits of *N*-salicylideneaniline at room temperature (RT), 443 K, 453 K, and 463 K during heating, and again at RT after cooling. Experimental diffraction data are shown as black dots, the calculated patterns as blue lines, and the difference curves in red. Vertical markers indicate the positions of Bragg reflections for Forms I (green), III (peach), and IV (teal). Hot-stage microscope images of the sample at 440 K and 460 K are shown alongside the diffraction patterns, highlighting the crystal jumping behavior observed in this temperature range.

During the first heating cycle, a prominent endothermic peak is observed with the onset around 446 K (~173 °C) and peaking around 448 K (~175 °C), indicating a phase transition likely associated with the thermosalient effect. This event is sharp and well-defined, typical for a first-order phase transition. No preceding exotherm is observed, and variable-temperature PXRD confirms that the starting material is fully crystalline (Forms I + III). We therefore exclude cold crystallization and assign the endotherm to the enantiotropic I/III → IV transition that also produces the thermosalient motion observed by hot-stage microscopy data (Figure 2). Upon the subsequent first cooling run, an exothermic peak occurs at a significantly lower temperature with the onset around 394 K (~125 °C) (peking around 396 K (~123 °C)), suggesting a hysteresis typical for kinetically hindered phase transformations. Such hysteresis is typical of all thermosalient phase transitions. This peak indicates recrystallization or structural rearrangement back into one of the lower-temperature polymorphs. PXRD collected at room temperature after cooling shows only Form III; Form I is absent.

The second heating cycle again reveals an endothermic peak at a similar temperature, with the onset around 441 K (~168 °C) (peaking around 444 K (~171 °C)), closely resembling the first heating event but with reduced enthalpy and a slightly broader profile. Because the bulk sample is now 100% Form III—the polymorph that carries the higher microstrain (see detailed discussion later in the text)—strain is distributed heterogeneously throughout the material. Domains therefore reach the III → IV instability at slightly different temperatures, broadening the DSC signal while the total ΔH decreases (the contribution from the I → IV pathway is no longer present). The second cooling trace shows a weaker, slightly shifted exotherm with the onset around 411 K (~140 °C) (peaking around 406 K (~133 °C)). The higher crystallization temperature in this cycle likely reflects residual nuclei, enhanced nucleation sites, or structural defects produced by the first heating run. Such behavior emphasizes the sensitivity of polymorphic crystallization pathways in salicylideneaniline to thermal history, underscoring the interplay between structural defects, nucleation kinetics, and crystallization temperature. Overall, the DSC data confirm that the thermosalient transitions are reproducible yet accompanied by thermal hysteresis and partial structural irreversibility. These results strongly support the presence of kinetically controlled polymorphic transformations and underline the complex interplay between thermal history, polymorph stability, and mechanical responsiveness in this thermosalient system.

Temperature-induced structural changes in *N*-salicylideneaniline were investigated by in situ variable X-ray powder diffraction (VTXRPD) in the temperature range of room temperature (RT, 298 K) to 493 K. This interval was chosen to include wide enough temperature ranges before and after the thermosalient phase transition [29] to obtain reliable data about structural changes leading to and after the phase transition. The Le Bail refinements of XRD patterns at selected temperatures are shown in Figure 2. The atomic models (including C, N, O, H positions) for each polymorph at room temperature were taken directly from Mittapalli et al. (2017) [29]. Because powder XRD cannot reliably refine light-atom positions or small bond-angle changes, all atomic coordinates other than unit-cell parameters and isotropic displacement factors were held fixed during the Le Bail refinement. Differences in crystal packing, bond distances, and bond angles among Forms I, III, and IV have been established in detail by Mittapalli et al. (2017) via single-crystal XRD [29]. Our powder diffraction data are not of sufficient resolution to re-derive those same atomic-level changes (especially H-atom coordinates). Instead, we focus on the more robust outputs—unit-cell evolution, principal thermal strains, and microstrain broadening—which directly reveal how lattice-scale stress accumulates and triggers thermosalient motion.

As depicted in Figure 2, the as-prepared sample consists of Forms I and III at RT in coexistence. This observation is extremely beneficial as it facilitates a direct comparison of the thermal behaviors of Forms I and III under identical experimental conditions. It is evident that the two polymorphic forms coexist until approximately 453 K, at which point the transformation of both phases into Form IV commences. Notably, at this temperature, all three phases (Form I, Form III, and Form IV) are present simultaneously. The phase transition concludes prior to 463 K, resulting in the sole presence of Form IV at that temperature. This observation is fully in accordance with the DSC data presented in Figure 1. Upon cooling, the sample converts to Form III (not back to the original Form I), indicating that Form III can be kinetically favored on recrystallization even if Form I was originally present. This aligns with the observed hysteresis (Figure 1) and suggests Form III is a metastable polymorph at room temperature that nucleates more readily under the given conditions. All the phase transformations during heating and cooling runs are in accordance with the findings of a previous study [29]. The thermal behavior of *N*-salicylideneaniline was monitored using hot-stage microscopy (Figure 1). During the first heating run, the vast majority of crystals exhibited forceful and energetic jumps in the temperature range of 440–460 K. No evidence of the thermosalient effect was observed during the subsequent cooling run. In the second heating cycle, the thermosalient effect reappeared near the phase transition between Form III and Form IV (approximately 444 K), although with markedly reduced intensity—both in the magnitude of the jumps and in the number of crystals displaying this behavior. 

**Figure 3:**
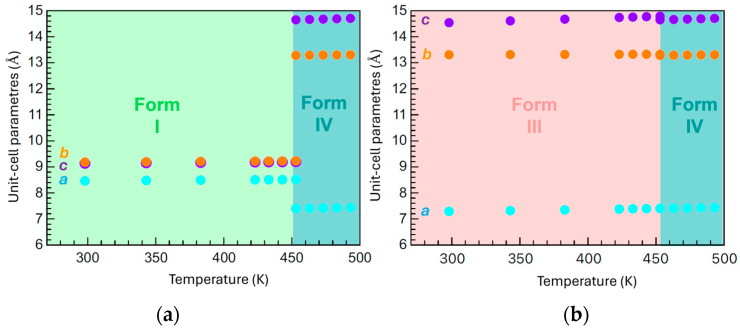
Temperature dependence of the unit-cell parameters (a, b, and c) for different polymorphs of *N*-salicylideneaniline. (**a**) shows Forms I and IV, while (**b**) shows Forms III and IV. All data points are represented as circles: cyan for the *a*-axis, orange for the *b*-axis, and violet for the *c*-axis. The phase transition to Form IV occurs near 453 K. Colored background regions indicate the stability ranges of each polymorph. Error bars are smaller than the symbol size.

Thermal expansion behavior of the three polymorphs of *N*-salicylideneaniline (Forms I, III, and IV) was systematically investigated by tracking the evolution of crystallographic lattice parameters using variable-temperature X-ray powder diffraction, as presented in Figure 3. A table listing the refined cell parameters (a, b, c, α, β, γ) and a volume of the unit cell for all three forms at all temperatures where diffraction data were collected is shown in the Supplementary Materials (Table S1). All three forms exhibit positive thermal expansion, indicating that the unit-cell volumes increase with rising temperature. However, significant differences in the anisotropy and nature of this expansion clearly distinguish the thermal responses of each polymorph and correlate with their respective structural stability and mechanical behavior.

Form I exhibits highly anisotropic thermal expansion, rationalized by directional intermolecular interactions and the inherent elastic anisotropy of its lattice. According to the crystal structure reported by Mittapalli et al., strong N–H···O and C–Cl···O hydrogen bonds are oriented primarily along the *c*-axis, while comparatively weaker π–π stacking interactions extend along the *b*-axis, and van der Waals contacts dominate along the *a*-axis [29]. This structural anisotropy is in full agreement with our findings: the *c*-axis, reinforced by strong hydrogen bonding, shows the smallest thermal expansion (Δ*c* ≃ +0.528%), whereas the *b*-axis, governed by weaker π–π interactions, expands the most (Δ*b* ≃ +0.651%), closely followed by the more compliant van der Waals-dominated *a*-axis (Δ*a* ≃ +0.612%). Consequently, lattice parameters evolve unevenly (Δ*c* < Δ*a* < Δ*b*), reflecting the interplay between strong hydrogen-bond reinforcement along *c*, moderate π–π constraints along *b*, and more compliant van der Waals slip-planes along *a*.

The thermal strain accumulates gradually until, near 450 K, Form I undergoes a sharp and discontinuous structural transition to Form IV. This transition is abrupt in nature, as seen by sudden jumps in the axial dimensions and angles, and it is characteristic of a thermosalient phase transition—an event where the accumulated internal strain is rapidly released, often resulting in macroscopic mechanical effects such as crystal jumping or fragmentation. Quantitative analysis of the lattice parameter changes reveals the transformation strain associated with this transition to be remarkably large and anisotropic. Specifically, the lattice contracts by approximately 13% along the *a*-axis, while expanding by 44% along the *b*-axis and an exceptional 59% along the *c*-axis. These drastic, directionally unequal changes reflect a large amount of internal elastic energy stored in the crystal lattice prior to the transition, which is consistent with the energy release mechanism driving thermosalient behavior.

In contrast, Form III exhibits very different thermal expansion behavior. Across the entire temperature range, the expansion of the lattice parameters is smooth and continuous, with moderate anisotropy among the axes. Although the *c*-axis shows slightly larger thermal responsiveness, the overall strain accumulation is more evenly distributed and does not exhibit sudden discontinuities. This gradual evolution indicates that Form III maintains structural integrity and adapts to thermal stress in a more cooperative and reversible manner. Despite the absence of visible discontinuity in Figure 3, it is important to note that Form III also exhibits thermosalient behavior. This suggests that even in the absence of dramatic lattice jumps, sufficient localized or anisotropic strain may still accumulate to trigger mechanical motion, albeit possibly through more subtle structural mechanisms than those observed in Form I.

Form IV, the high-temperature polymorph that emerges after the phase transition from either Form I or III, displays a much more isotropic and restrained thermal expansion profile. All three crystallographic axes increase moderately and nearly equally with temperature, and the unit-cell angles remain relatively stable. This indicates a relaxed and mechanically stabilized lattice configuration in the post-transition phase. The near-isotropic expansion implies that the internal stress has been largely dissipated during the transition, and the resulting structure is less prone to mechanical instability or further structural transformation within the studied temperature range. Overall, the data presented in Figure 3 clearly demonstrates that Forms I and III, while both capable of exhibiting thermosalient transitions, do so via different strain accumulation and release mechanisms. Form I undergoes a classic, strain-driven thermosalient transition marked by drastic lattice deformation and discontinuity, whereas Form III accumulates thermal strain more gradually, potentially relying on subtler internal distortions to drive its mechanical response. Form IV, in contrast, represents a relaxed structural endpoint, underscoring the link between lattice anisotropy, strain dynamics, and phase transition mechanisms in thermosalient molecular crystals.

While the crystallographic data provided valuable insights into anisotropic expansion, it failed to reveal any negative thermal expansion (NTE) behavior typically associated with mechanical instability and thermosalient phenomena. This limitation demanded a more detailed tensorial approach to fully capture the complexity of the lattice deformation. Analysis based solely on crystallographic parameters inherently overlooks shear strains and angular distortions within the lattice. As such, it may mask internal contraction mechanisms crucial for understanding structural instability. Therefore, principal axis thermal expansion analysis was performed to gain a complete picture of the crystal’s mechanical response to heating.

While the crystallographic data provided valuable insights into anisotropic expansion, it failed to reveal any negative thermal expansion (NTE) behavior typically associated with mechanical instability and thermosalient phenomena. This limitation demanded a more detailed tensorial approach to fully capture the complexity of the lattice deformation. Analysis based solely on changes in the *a*, *b*, and *c* parameters inherently overlooks shear strains and angular distortions within the lattice, potentially masking internal contraction mechanisms that are crucial for understanding structural instability. Instead of only describing expansion along the crystallographic axes, we therefore calculated the principal axes of expansion by diagonalizing the thermal strain tensor. In practical terms, this involves finding the set of orthogonal directions in the crystal that correspond to the maximum, intermediate, and minimum expansion. For a low-symmetry unit cell such as in this system, the directions of greatest expansion do not necessarily coincide with the *a-*, *b-*, or *c*-axis. By determining these principal expansion axes, we can, for example, state that the crystal stretches predominantly along a direction lying within a particular crystallographic plane, rather than juggling separate changes along oblique cell axes. This tensorial analysis makes it far easier to visualize and compare anisotropic expansion across different polymorphs: one can imagine the crystal stretching most along a single principal direction, with less deformation along the other two, rather than trying to interpret correlated changes in three skewed lattice vectors. To investigate the thermal behavior of principal axis analysis and to gain a complete picture of the crystal’s mechanical response to heating, the PASCal software was utilized, which provided significant additional insights [30]:

1. Form I showed a significant negative thermal expansion along principal axis *X*_1_ (−10.0324(13) × 10^−6^ K^−1^), combined with a large positive expansion along *X*_3_ (+136.0098(42) × 10^−6^ K^−1^). This tensorial analysis uncovered internal contraction previously hidden by crystallographic measurements.
2. Form III exhibited anisotropic but positive expansion along all principal axes (*X*_3_ = +153.3505(160) × 10^−6^ K^−1^, *X*_2_ = +48.3779(105) K^−1^× 10^−6^, *X*_1_ = +1.5323(20) × 10^−6^ K^−1^).
3. Form IV showed uniformly positive principal expansions, consistent with structural relaxation after the thermosalient event.

The thermal expansion coefficients *α* for Forms I, III, and IV along the principal axis *i* = 1, 2, 3 are shown in Table 1.

**Figure 4:**
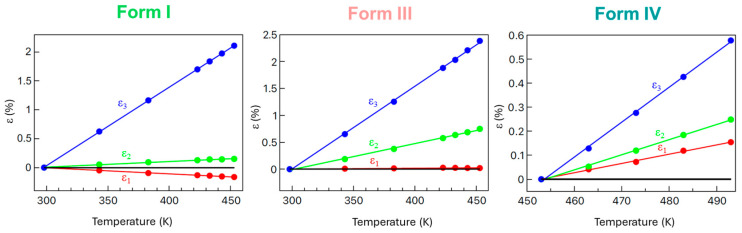
Relative strain along the principal axes *X*_1_, *X*_2_, and *X*_3_ for Forms I, III, and IV of *N*-salicylideneaniline, obtained from PASCal analysis. The plots reveal strong anisotropy and the presence of negative thermal strain in Form I. The horizontal black line at 0% marks the threshold separating expansion (positive strain) from contraction (negative strain), helping visualize the direction and magnitude of principal axis deformation. Error bars are smaller than the symbol size.

**Figure 5:**
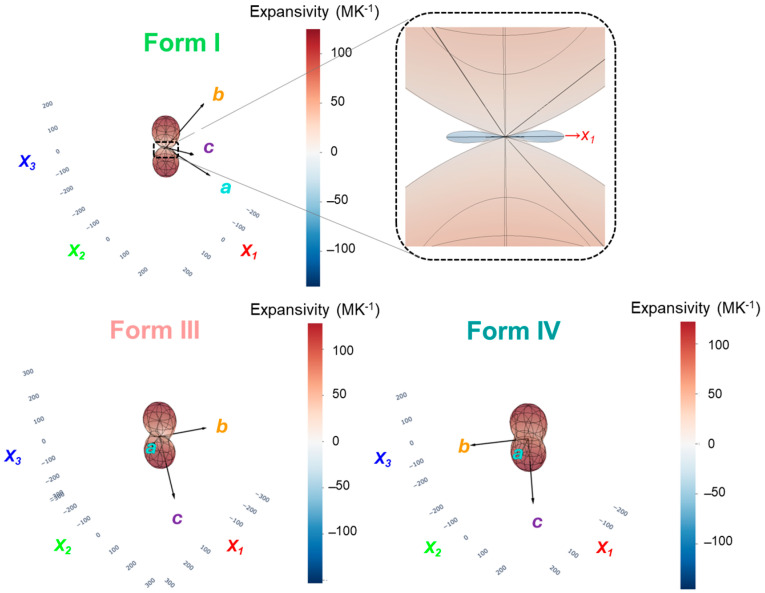
Thermal expansion indicatrix plots for Forms I, III, and IV obtained through principal axis analysis. The 3D expansivity surfaces (strain ellipsoids) visually represent the anisotropy of thermal expansion across the three polymorphs. Axes are labeled by both the crystallographic directions (*a*, *b*, *c*) and the principal axes of expansion (*X*_1_, *X*_2_, *X*_3_). Colors represent the magnitude and sign of linear thermal expansivity: red for positive expansion and blue for negative expansion, in units of MK^−1^ (10^−6^ K^−1^). The top-right inset shows an enlarged view of the negative thermal expansion along principal axis *X*_1_ in Form I, which is not easily visible at the original scale.

These results emphasize that tensorial strain analysis is necessary to detect directional contractions hidden within overall positive volumetric expansion. Figure 4 illustrates the relative strain along the principal axes *X*_1_, *X*_2_, and *X*_3_ for Forms I, III, and IV, obtained from PASCal analysis. Figure 5 depicts the thermal expansion indicatrix for Forms I, III, and IV.

Although the crystallographic lattice parameters (a, b, c) of Form I show consistent positive thermal expansion with increasing temperature, a more complete understanding of the material’s mechanical response is obtained through tensorial analysis. Application of the PASCal software, which performs eigenvalue decomposition of the full strain tensor derived from variable-temperature crystallographic data, reveals a negative thermal expansion (NTE) coefficient along the principal axis *X*_1_. This finding appears counterintuitive, as it suggests contraction along one spatial direction despite uniform expansion along all unit-cell axes. This apparent contradiction is reconciled by recognizing that the principal axes of expansion—*X*_1_, *X*_2_, and *X*_3_—are not constrained to align with the crystallographic axes but rather correspond to the eigenvectors of the thermal expansion tensor. These directions reflect the true, net deformation behavior of the lattice, including contributions from angular strain (changes in α, β, γ) and shear deformation. In triclinic or otherwise low-symmetry crystals, the deformation associated with heating is often highly anisotropic, and the volumetric expansion can be redistributed such that contraction occurs along a diagonal or off-axis direction.

In the case of Form I, the PASCal analysis yielded principal expansion coefficients of *α*_1_ = –10 × 10^−6^ K^−1^, *α*_2_ = +10 × 10^−6^ K^−1^, and *α*_3_ = +136 × 10^−6^ K^−1^. While the expansion along *X*_3_ dominates, the contraction along *X*_1_—despite positive Δ*a*, Δ*b*, and Δ*c*—indicates that cooperative motion, such as molecular tilting or hinge-like flexibility in the packing motif, results in net shrinkage along this eigenvector. This behavior is consistent with the structural complexity of molecular solids and reinforces the need to go beyond scalar lattice parameter analysis when interpreting mechanical and thermal responses.

Analysis of relative strain along the principal axes provides critical mechanistic insight into the origin of the thermosalient effect in Form I. As shown in Figure 5, heating induces highly anisotropic deformation: a significant positive strain accumulates along principal axis X_3_, moderate expansion occurs along X_2_, while strain along X_1_ remains close to zero or becomes slightly negative. This anisotropic strain distribution leads to internal stress buildup, especially in directions constrained from expanding freely. The near-zero or negative thermal strain along X_1_ implies contraction or structural frustration occurring along this direction, while the other axes expand. Such directional mechanical frustration acts as a precursor to lattice instability and sudden mechanical actuation. In this context, the presence of negative thermal expansion (NTE) along a principal axis is particularly important—it reflects the redistribution of volumetric expansion into contraction along a non-crystallographic direction. This promotes localized stress accumulation, which, when exceeding the mechanical limits of the structure, results in the rapid release of elastic energy as observed in thermosalient behavior. These findings highlight the necessity of full tensorial thermal expansion analysis to reveal hidden structural responses that are not detectable from crystallographic parameters alone and to understand the coupling between microscopic strain evolution and macroscopic mechanical output in responsive molecular crystals. Our PASCal analysis revealed significant NTE along a principal axis in Form I, highlighting directional strain accumulation as the mechanistic underpinning for abrupt mechanical release. This contrasts markedly with Form III, where distributed microstrain, evidenced by subtle but progressive changes in fingerprint plots, accumulates gradually, leading to a smoother actuation. While the pronounced anisotropic strain and negative thermal expansion observed in Form I clearly explain its thermosalient behavior, Form III exhibits more moderate and isotropic expansion, suggesting that thermal strain alone may not fully account for its mechanical response.

**Figure 6:**
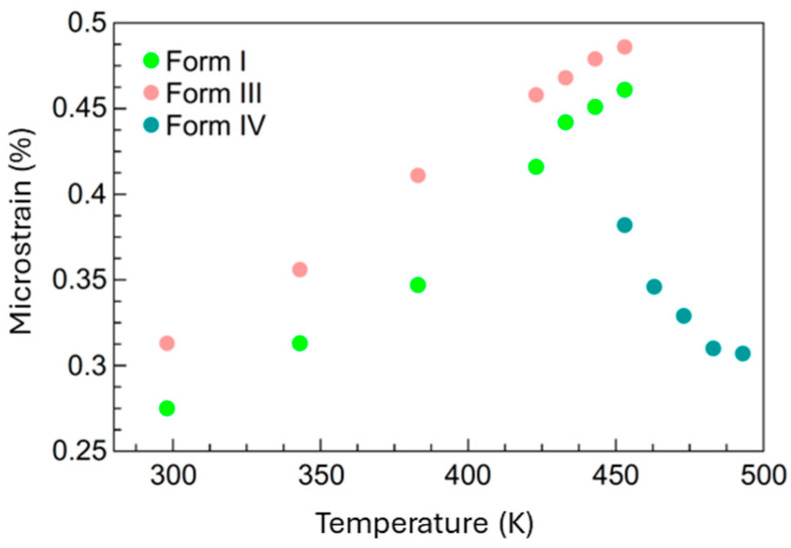
Temperature dependence of microstrain for Forms I, III, and IV. The microstrain values illustrate the differences in internal strain accumulation among the polymorphs and clearly highlight the differences in strain evolution among the polymorphic forms across the temperature range from RT to 500 K. Temperature dependence of microstrain in Forms I, III, and IV. The plot reveals distinct trends in internal strain accumulation for each polymorph over the temperature range from room temperature to 500 K. Differences in the magnitude and evolution of microstrain reflect structural variations and thermal responses unique to each form. Data points are color-coded by polymorph: green for Form I, peach for Form III, and teal for Form IV.

To further explore the origins of the thermosalient effect—particularly in cases where macroscopic anisotropy is less extreme—we extended our analysis by calculating microstrain for all three forms (I, III, and IV). Microstrain refers to localized, small-scale distortions within the crystal lattice, often arising from defects, dislocations, or non-uniform internal stresses. Unlike thermal strain, which reflects average macroscopic deformation, microstrain captures subtle structural inhomogeneities that may not manifest in overall lattice parameter changes. It is quantitatively expressed as a dimensionless root mean square (RMS) value of the relative variation in lattice spacing (Δ*d*/*d*), representing the statistical distribution of interplanar distances across the crystal [31]. In this study, microstrain was quantitatively assessed by analyzing the broadening of diffraction peaks using the strain analysis module in X’Pert HighScore Plus software which applies a Caglioti-based profile fitting approach [32]. This approach allowed us to isolate the strain contribution from instrumental and crystallite size effects, providing a reliable measure of internal stress evolution across Forms I, III, and IV and thus to gain deeper insight into additional mechanisms contributing to the dynamic behavior of this system. The progression of microstrain with temperature is shown in Figure 6.

The microstrain values depicted in Figure 6 clearly illustrate differences in thermal behavior across the polymorphs (Forms I, III, and IV) and provide key insights into their thermosalient transitions. Upon careful inspection, Form I shows an increase in microstrain from approximately 0.30% at 300 K to about 0.45% near 450 K, while Form III similarly experiences a comparable absolute increase in microstrain—from roughly 0.35% to nearly 0.50% within a similar temperature range. Notably, Form III consistently exhibits higher absolute microstrain values across the temperature range compared to Form I, despite their comparable overall increases (both approximately 0.15%). This observation—Form III having slightly higher absolute strain values—implies that the internal elastic energy accumulation in Form III is at least as significant as that in Form I. Given experimental evidence that both polymorphs demonstrate strong thermosalient behavior (Videos S1 and S2 in the Supplementary Materials, also observed by [29]), this suggests that the magnitude of the absolute microstrain is indeed strongly correlated with their thermosalient activity. Higher absolute strain values can enhance the internal energy stored, making Form III particularly efficient in translating accumulated elastic energy into mechanical motion upon structural transitions. The similar trends in microstrain evolution (parallel increases with temperature) observed for Forms I and III support the conclusion that both polymorphs accumulate elastic strain effectively, thus reinforcing their thermosalient character. The subtle difference in absolute values (slightly higher in Form III) suggests that Form III might possess structural anisotropies or lattice features which allow even greater internal elastic energy storage prior to transition. In contrast, Form IV clearly demonstrates decreasing microstrain above approximately 450 K. This decline indicates a structural relaxation and the subsequent reduction in internal stress and elastic energy post-transition, thereby marking the cessation of thermosalient activity. Thus, it can be concluded that microstrain represents an important and previously underexplored mechanism contributing to the thermosalient effect. This study highlights microstrain as a complementary and independent stress pathway beyond macroscopic lattice anisotropy, revealing a novel feature in the understanding of dynamic mechanical responses in molecular crystals.

To conduct a comprehensive analysis of thermosalient behavior in the polymorphs of *N*-salicylideneaniline, Hirshfeld surface analysis was performed utilizing the CrystalExplorer program [33]. While thermal expansion and microstrain measurements revealed how structural distortions evolve with temperature and accumulate internal elastic energy, Hirshfeld surface analysis provided complementary insights at the molecular interaction level. By quantifying the nature and strength of intermolecular contacts across different polymorphs and temperatures, this method enabled the identification of specific interaction motifs—such as π–π stacking, hydrogen bonding, and van der Waals forces—that either resist or facilitate deformation. This interaction-based perspective is crucial to understanding how localized stresses arise and propagate through the crystal lattice, ultimately leading to the abrupt mechanical responses observed in thermosalient transitions. Together, these multiscale analyses form a coherent picture of how structural anisotropy, strain buildup, and intermolecular interactions collectively govern the thermosalient behavior in this system. Hirshfeld surface fingerprint plots provide crucial insights into the nature and distribution of close intermolecular contacts in the crystal lattice. In the case of *N*-salicylideneaniline, these plots reveal how local interaction geometries evolve with temperature, offering a structural basis for understanding the distinct mechanical responses of different polymorphic forms. This analysis supports the presence of two fundamentally different thermosalient mechanisms operating in this system: an abrupt, strain-release mechanism in Form I, and a gradual, microstrain accumulation mechanism in Form III.

**Figure 7:**
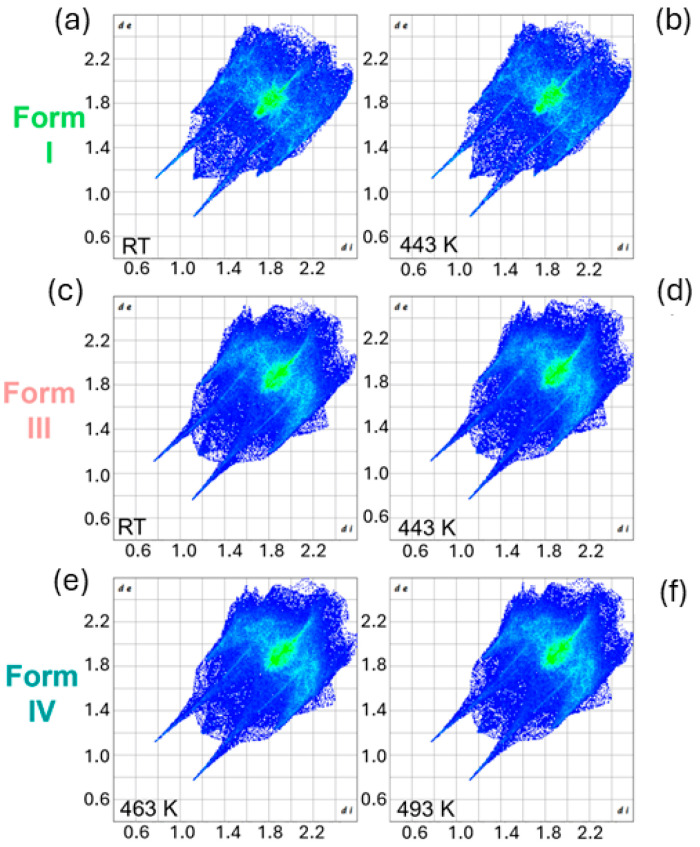
Hirshfeld surface fingerprint plots for three polymorphs of *N*-salicylideneaniline at different temperatures (**a**–**f**). Each row corresponds to a distinct polymorphic form: Form I, Form III, and Form IV. For each form, plots are shown before and after heating to the indicated temperatures. The plots visualize the evolution of intermolecular contact patterns across the thermosalient transitions.

At room temperature, the fingerprint plot of Form I (Figure 7a) is sharply defined, with narrow and intense spikes. These correspond to short Cl···H and Cl···O contacts close to van der Waals limits. The central green region is tightly clustered, reflecting a constrained, highly directional packing network. This structural rigidity offers no pathway for local adjustment under thermal stress, which instead accumulates until released in an abrupt mechanical event—a hallmark of the strain-driven thermosalient mechanism. In contrast, Form III at room temperature (Figure 7b) exhibits a broader, more diffuse fingerprint profile. The spikes are less pronounced, and the central region is more expansive, indicating a more heterogeneous and relaxed contact environment. This packing arrangement provides the lattice with local flexibility to accommodate thermal motion through subtle rearrangements. Such behavior supports the microstrain-driven thermosalient mechanism, wherein structural stress is dispersed and builds incrementally across many contact types until reaching a transition threshold. Upon heating, both polymorphs undergo fingerprint evolution that further supports the dual-mechanism model. In Form I (Figure 7c), contact features persist with only slight softening of spikes and minimal broadening of the central region. This lack of contact redistribution implies that the accumulated strain is not dissipated progressively but instead builds directionally until it exceeds the elastic limit, triggering the sudden thermosalient event. In contrast, the fingerprint plot of Form III at 443 K (Figure 7d) reveals more evident changes: spikes spread and soften, and the central region shows greater dispersion. These features indicate a more responsive lattice that incrementally adapts to thermal expansion. Internal distortions accumulate gradually and distribute across the structure, eventually resulting in a mechanical response that is smoother and less abrupt than in Form I. Finally, the plots for Form IV at 463 K and 493 K (Figure 7e,f) remain essentially unchanged, demonstrating that this phase is thermally inert and mechanically relaxed. The absence of fingerprint evolution indicates the conclusion of both transformation pathways—regardless of whether initiated by directional strain (Form I) or accumulated microstrain (Form III).

Quantitative analysis of contact percentages, shown in Table 2, reinforces these conclusions. In Form I, contributions remain nearly static from room temperature to 443 K—for example, Cl···H increases only slightly from 29.4% to 29.6%, while C···C decreases from 13.0% to 12.8%. This confirms that thermal stress in Form I does not manifest through widespread contact evolution but is instead funneled into mechanical strain, consistent with a strain-dominated response. In Form III, several contact types adjust with heating. Cl···H increases from 23.2% to 23.6%, H···H from 19.3% to 19.4%, and O···H decreases from 11.2% to 10.8%. These small but distributed changes suggest the progressive buildup of internal distortions—the fingerprint signature of a microstrain-driven mechanism. For Form IV, contact percentages between 463 K and 493 K remain stable, confirming this phase as the relaxed endpoint of both thermosalient pathways. Its invariance in contact structure supports the idea that the system has reached a mechanically neutral state.

Taken together, these fingerprint and contact data establish a clear distinction between the strain-driven transition in Form I, marked by structural rigidity and abrupt mechanical release, and the microstrain-mediated behavior in Form III, which involves gradual adaptation and distributed contact evolution. Both pathways ultimately lead to the same relaxed Form IV phase but differ fundamentally in how they accommodate and release internal stress—a key insight for understanding and designing thermoresponsive materials.

## 3. Materials and Methods

### 3.1. Experimental Techniques

The title compound was synthesized following the procedure reported by Mittapalli et al. [29]. A total of 1 mmol of 4-aminobenzamide was dissolved in 8 mL of anisole in a 100 mL round-bottom flask within a Dean–Stark apparatus. To this solution, 1 mmol of 3,5-dichlorosalicylaldehyde was added, and the mixture was refluxed for 6 h. The resulting red crystalline product was isolated and washed thoroughly with n-hexane (7 times) to remove any unreacted starting materials or by-products.

### 3.2. Variable-Temperature X-Ray Powder Diffraction

Temperature-induced structural changes were tracked by in situ HT variable-temperature (VT) XRPD using a Philips PW 1710 diffractometer (Philips, Almelo, The Netherlands) equipped with a high-temperature chamber. Diffraction patterns were collected in a 2θ range of 5–40° with a step size of 0.02° and a measuring time of 1 s/step using monochromatized Cu*K*α radiation (monochromator: graphite). Data were collected in a temperature range of 300 K–495 K. Crystal structures were refined, and microstrain analysis was conducted by the Le Bail method using HighScore X’pert Plus (Version 4.5, March 2016). Refinement was conducted by using the split-type pseudo-Voigt profile function and the polynomial background model. During the refinement, unit-cell parameters, zero shift, half-width parameters, asymmetry, and peak shape parameters were simultaneously refined. The structure of *N*-salicylideneaniline Forms I and III, as reported by [29], was used as the starting structural model for the Le Bail refinement of data collected at temperatures up to (and including) 453 K, while the structure *N*-salicylideneaniline Form IV, as reported by [29], was used for the Le Bail refinement of data collected at higher temperatures (453–493 K). Thermal expansion coefficients were calculated from the refined unit-cell parameters obtained from variable-temperature diffraction data. Linear axial thermal expansion coefficients along the principal axes were calculated using the PASCal software [30].

### 3.3. Thermal Analysis

Differential scanning calorimetry (DSC) was carried out using a Mettler Toledo DSC 822e instrument (Mettler-Toledo International Inc., Switzerland) equipped with an FRS5+ sensor containing 56 AuPd thermocouples, ensuring high sensitivity and temperature resolution. Measurements were performed in a dynamic nitrogen atmosphere (flow rate 50 mL/min) over a temperature range of 293–483 K. Approximately 1100 mg of sample was sealed in standard 40 μL aluminum crucibles and subjected to heating and cooling cycles at a rate of 10 °C/min under identical thermal conditions.

### 3.4. Hirshfeld Surface and Intermolecular Contact Analysis

Hirshfeld surface analyses were performed using CrystalExplorer (Version 21.5, 2017) for three polymorphs of *N*-salicylideneaniline [33]. The analyses were carried out at two temperatures for each form: Form I and Form III were analyzed at room temperature and at 170 °C, while Form IV was analyzed at 190 °C and 220 °C. Hirshfeld surfaces were generated using standard parameters based on *d_norm_*, and the corresponding 2D fingerprint plots were used to quantify the relative contributions of specific intermolecular contacts.

### 3.5. Hot-Stage Microscopy

Mechanical behavior during heating/cooling was recorded using a Nikon Eclipse LV150NL (Nikon, Tokyo, Japan) optical microscope equipped with a Linkam THMS600 (Linkam Scientific Instruments, Salfords, Surrey, UK) hot-stage and OPTOCAM-II(Optolution GmbH, Lörrach, Baden-Württemberg, Germany) color camera with a resolution of 1600 × 1200 pixels. Crystal behavior was monitored in the temperature interval from room temperature to the melting point (523 K).

## 4. Conclusions

This study offers a detailed structural and mechanistic analysis of the thermosalient behavior in *N*-salicylideneaniline, establishing the existence of two fundamentally distinct phase transition pathways within a single molecular system. By integrating variable-temperature X-ray powder diffraction, differential scanning calorimetry, thermal expansion tensor analysis, microstrain calculations, and Hirshfeld surface fingerprinting, we demonstrate that Forms I and III of this compound respond to thermal stimuli through different mechanisms, both converging into the relaxed high-temperature Form IV. Form I undergoes a sharp and explosive thermosalient transition, driven by the accumulation of pronounced anisotropic strain along specific crystallographic axes. This is evidenced by extremely high directional thermal expansion, including negative expansion along one principal axis, and by a rigid molecular packing that shows negligible rearrangement of contact interactions with heating. The inability of the lattice to redistribute thermal stress progressively leads to sudden mechanical actuation, consistent with a strain-driven transition model. In contrast, Form III exhibits a more gradual, microstrain-based phase transition. Its fingerprint plots reveal broader features and more dynamic evolution of contact types with temperature, indicating a more conformationally adaptable packing. Small but measurable shifts in interaction contributions, particularly in Cl···H, H···H, and O···H contacts, suggest an incremental buildup of internal distortions. This is further supported by an increase in microstrain prior to the phase transition, suggesting that mechanical actuation is initiated not by sudden lattice collapse, but by the culmination of locally accumulated stress. The resulting transformation is less abrupt, structurally smoother, and qualitatively different from the behavior of Form I. The final polymorph, Form IV, displays remarkable thermal and mechanical stability. Across the high-temperature regime, its lattice parameters, microstrain levels, and Hirshfeld contact profiles remain essentially unchanged. This confirms its role as a relaxed, non-responsive phase into which both the strain-driven and microstrain-driven pathways ultimately converge. This work demonstrates the importance of considering both macroscopic and microscopic stress metrics in the analysis of thermoresponsive materials. Whereas thermal strain describes bulk anisotropy in expansion behavior, microstrain reveals the accumulation of internal deformation at the local level. By leveraging these complementary measures alongside molecular contact analysis, we establish a framework for understanding and differentiating thermosalient responses based on structural flexibility and interaction dynamics. The findings presented here expand the conceptual boundaries of thermosalient phase transitions. Our findings reveal that the thermosalient effect in this polymorphic system can originate from two fundamentally distinct mechanisms—one driven by abrupt lattice strain release reminiscent of a quasi-martensitic transition, and the other by the gradual buildup of microstrain and defect formation. The explicit recognition of two coexisting mechanistic pathways within the same compound, combined with the introduction of microstrain as a critical driving factor, provides a new perspective on how molecular crystals can be engineered to exhibit tunable mechanical actuation. This dual-mechanism model opens exciting possibilities for the design of smart materials capable of directional, temperature-triggered motion, and sets the stage for further exploration of controlled responsiveness in molecular solids.

## Figures and Tables

**Table 1:** Thermal expansion coefficients *α_i_* along principal axis *X_i_* (*i* = 1, 2, 3) for Forms I, III, and IV determined from in situ VTXRD.

			Component of *x_i_* Along the Crystallographic Axes		
	Principal Axis	*α_i_* ( $\times 10^{-6}K^{-1}$)	*a*	*b*	*c*
Form I	*X*_1_	−10.0324(13)	0.4724	0.7403	0.4783
	*X*_2_	10.2472(26)	0.6276	−0.0679	0.7755
	*X*_3_	136.0098(42)	0.6997	−0.5587	−0.4452
Form III	*X*_1_	21.5323(20)	0.2675	0.924	−0.2732
	*X*_2_	48.3779(105)	0.8894	−0.2699	−0.3689
	*X*_3_	153.3505(160)	0.8764	−0.035	04803
Form IV	*X*_1_	38.5886(40)	0.674	−0.6455	−0.3593
	*X*_2_	62.8973(120)	0.7161	0.4656	−0.5199
	*X*_3_	145.3516(227)	0.9432	−0.846	0.3212

**Table 2:** Hirshfeld surface contact contributions (% surface area) for Forms I, III, and IV of *N*-salicylideneaniline at relevant temperatures.

Interaction Type	Form I RT (%)	Form I 443 K (%)	Form III RT (%)	Form III 443 K (%)	Form IV 463 K (%)	Form IV 493 K (%)
Cl···H	29.4	29.6	23.2	23.6	23.3	23.3
H···H	18.3	18.3	19.3	19.4	19.4	19.4
O···H	12.9	12.9	11.2	10.8	11.9	11.9
Cl···Cl	7.4	7.4	8.4	8.3	7.6	7.6
Cl···O	2.1	2.2	1.5	1.6	2.1	2.1
C···C	13.0	12.8	10.4	10.4	8.0	8.0
C···H	10.4	10.4	13.2	13.5	13.1	13.1
H···C	5.0	5.0	17.3	17.1	15.9	15.9
Cl···N	0.7	0.7	1.3	1.2	1.0	1.0

## Data Availability

Data are contained within the article and Supplementary Materials.

## Supplementary Materials

The following supporting information can be downloaded at https://www.mdpi.com/article/10.3390/molecules30122567/s1, Videos S1 and S2: Hot-stage microscopy videos of *N*-salicylideneaniline recorded during heating at a rate of 10 K·min^−1^. Both videos capture the thermosalient effect, characterized by sudden and energetic crystal jumping in response to a phase transition. The majority of crystals exhibit this mechanical response within a narrow temperature range of approximately 20 K.
