# Supplementary material for: Thermal Strain and Microstrain in a Polymorphic Schiff Base: Routes to Thermosalience
Source: Molecules. 2025 Jun 12;30(12):2567. doi: 10.3390/molecules30122567 (PMC12196372; doi:10.3390/molecules30122567)
Supplement: Supplementary file 1 [file molecules-30-02567-s001.zip › molecules-3663819-supplementary.pdf]

Table S1. Unit Cell Parameters of Salicylideneaniline (Forms I, III, and IV)

	T(K)	298	343	383	423	433	443	453	463	473	483	493
Form I	*a* (Å)	8.461(7)	8.477(1)	8.490(4)	8.503(5)	8.506(6)	8.509(8)	8.512(8)				
	b (Å)	9.136(3)	9.154(8)	9.169(3)	9.184(5)	9.188(9)	9.191(8)	9.195(6)				
	c (Å)	9.182(2)	9.196(4)	9.208(3)	9.220(6)	9.224(4)	9.227(7)	9.230(5)				
	α (°)	81.4(2)	81.2(3)	81.0(7)	80.9(3)	80.8(9)	80.8(5)	80.8(2)				
	β (°)	76.9(1)	77.1(4)	77.2(9)	77.4(5)	77.4(9)	77.5(4)	77.5(8)				
	γ (°)	80.5(3)	80.8(2)	81.0(5)	81.2(9)	81.3(5)	81.(1)	81.4(7)				
	V (Å^3)	677.4(2)	681.7(1)	685.3(1)	688.9(2)	689.8(2)	690.8(1)	691.7(1)				
Form III	*a* (Å)	7.290(1)	7.319(1)	7.347(2)	7.379(1)	7.387(2)	7.396(2)	7.405(3)				
	b (Å)	13.310(1)	13.315(1)	13.319(3)	13.324(2)	13.325(2)	13.326(1)	13.327(1)				
	c (Å)	14.541(2)	14.611(1)	14.676(1)	14.740(1)	14.755(1)	14.772(2)	14.790(7)				
	α (°)	87.6(7)	87.5(7)	87.4(7)	87.3(7)	87.3(4)	87.3(1)	87.2(8)				
	β (°)	80.8(1)	80.5(3)	80.2(8)	80.0(2)	79.9(6)	79.8(8)	79.8(1)				
	γ (°)	75.5(8)	75.6(1)	75.6(3)	75.6(5)	75.6(6)	75.6(7)	75.6(8)				
	V (Å^3)	1348.9(1)	1360.4(2)	1371.2(1)	1382.8(1)	1385.6(1)	1388.7(2)	1391.9(1)				
Form IV	*a* (Å)							7.402(1)	7.410(1)	7.419(1)	7.429(0)	7.438(2)
	b (Å)							13.283(7)	13.289(6)	13.296(1)	13.302(1)	13.308(1)
	c (Å)							14.651(2)	14.663(2)	14.677(1)	14.691(1)	14.706(2)
	α (°)							87.5(2)	87.5(2)	87.5(1)	87.5(1)	87.5(2)
	β (°)							80.0(4)	80.0(1)	79.9(5)	79.9(1)	79.8(6)
	γ (°)							75.9(5)	75.9(5)	75.9(4)	75.9(4)	75.9(4)
	V (Å^3)							1376.4(2)	1379.5(3)	1382.8(1)	1386.5(1)	1390.0(2)

Videos S1 and S2: Hot-stage microscopy videos of *N*-salicylideneaniline recorded during heating at a rate of 10 K·min^{-1}. Both videos capture the thermosalient effect, characterized by sudden and energetic crystal jumping in response to a phase transition. The majority of crystals exhibit this mechanical response within a narrow temperature range of approximately 20 K.
